# Changes in the number of new takeaway food outlets associated with adoption of management zones around schools: A natural experimental evaluation in England

**DOI:** 10.1016/j.ssmph.2024.101646

**Published:** 2024-03-19

**Authors:** John Rahilly, Ben Amies-Cull, Michael Chang, Steven Cummins, Daniel Derbyshire, Suzan Hassan, Yuru Huang, Matthew Keeble, Bochu Liu, Antonieta Medina-Lara, Oliver Mytton, Nina Rogers, Bea Savory, Annie Schiff, Stephen J. Sharp, Richard Smith, Claire Thompson, Martin White, Jean Adams, Thomas Burgoine

**Affiliations:** a MRC Epidemiology Unit, University of Cambridge School of Clinical Medicine, Box 285 Institute of Metabolic Science, Cambridge Biomedical Campus, Cambridge, CB2 0QQ, UK; b Nuffield Department of Primary Care Health Sciences, University of Oxford, Oxford, UK; c Office for Health Improvement and Disparities, Department of Health and Social Care, UK; d Department of Public Health, Environments & Society, Faculty of Public Health & Policy, London School of Tropical Hygiene and Medicine, 15-17 Tavistock Place, London, WC1H 9SH, UK; e Department of Public Health and Sport Sciences, Faculty of Health and Life Sciences, University of Exeter, UK; f Great Ormond Street Institute of Child Health, University College London, UK; g School of Health and Social Work, University of Hertfordshire, UK; h Department of Urban Planning, College of Architecture and Urban Planning, Tongji University, Shanghai, China

**Keywords:** Takeaway management zones, Exclusion zones, Schools, Takeaways, Urban planning, Natural experiment, Interrupted time series

## Abstract

By the end of 2017, 35 local authorities (LAs) across England had adopted takeaway management zones (or “exclusion zones”) around schools as a means to curb proliferation of new takeaways. In this nationwide, natural experimental study, we evaluated the impact of management zones on takeaway retail, including unintended displacement of takeaways to areas immediately beyond management zones, and impacts on chain fast-food outlets. We used uncontrolled interrupted time series analyses to estimate changes from up to six years pre- and post-adoption of takeaway management zones around schools. We evaluated three outcomes: mean number of new takeaways within management zones (and by three identified sub-types: full management, town centre exempt and time management zones); mean number on the periphery of management zones (i.e. within an additional 100 m of the edge of zones); and presence of new chain fast-food outlets within management zones. For 26 LAs, we observed an overall decrease in the number of new takeaways opening within management zones. Six years post-intervention, we observed 0.83 (95% CI -0.30, −1.03) fewer new outlets opening per LA than would have been expected in absence of the intervention, equivalent to an 81.0% (95% CI -29.1, −100) reduction in the number of new outlets. Cumulatively, 12 (54%) fewer new takeaways opened than would have been expected over the six-year post-intervention period. When stratified by policy type, effects were most prominent for full management zones and town centre exempt zones. Estimates of intervention effects on numbers of new takeaways on the periphery of management zones, and on the presence of new chain fast-food outlets within management zones, did not meet statistical significance. Our findings suggest that management zone policies were able to demonstrably curb the proliferation of new takeaways. Modelling studies are required to measure the possible population health impacts associated with this change.

## Highlights

• LAs in England can adopt takeaway management (“exclusion”) zones around schools.
• We evaluated the impacts of this intervention on takeaway retail using ITS analysis.
• For 26 LAs, we observed a decrease in the number of new takeaways within zones.
• Cumulatively, 12 (54%) fewer takeaways opened than anticipated after six years.
• Management zones were able to demonstrably curb the proliferation of new takeaways.

## 1 Introduction

Takeaway food outlets (“takeaways”) sell hot food for consumption off the premises (Jaworowska et al., 2014). Takeaway food tends to be energy-dense and nutrient poor (Jaworowska et al., 2014; Monsivais & Drewnowski, 2007; Robinson et al., 2018) and sold in large portions (Jaworowska et al., 2013). As a result, regular consumption of takeaway food is a public health concern. Frequent consumption has been associated with weight gain and obesity risk over time (Duffey et al., 2007; Penney et al., 2017). Diets of regular takeaway consumers tend to be higher in total energy, saturated fats, sugar and sodium (Jaworowska et al., 2014). Children and young adults consume more takeaway meals more frequently than those of other ages (Adams et al., 2015). In 2017 there were approximately 57,000 takeaways in England (Butler, 2017), but across the globe takeaways are also highly prevalent. Through making unhealthy choices the easy choice, there is emerging evidence that neighbourhood exposure to takeaways may be associated with takeaway food consumption (Jiang et al., 2023; Townshend & Lake, 2017). Moreover, recent growth in takeaway retail has been concentrated in deprived communities. This has resulted in a social gradient in exposure, which may contribute to observed health inequalities in the UK and elsewhere (Maguire at al., 2015; Public Health, 2018). In the UK and US, takeaways also cluster disproportionately around schools (Smith et al., 2013; Trapp et al., 2023), which may be implicated in the development of childhood obesity in these contexts.

Takeaways may be a modifiable risk factor for downstream health impacts (Lake et al., 2017). In the UK, to open a new takeaway, or to change the use of an existing premises to a new takeaway, planning permission must be sought from the local authority (LA). It is possible for this permission to be refused by urban planners, which can be on public health grounds (Keeble et al., 2019). By the end of 2017, 35 LAs across England had adopted takeaway management zones (sometimes referred to by LAs as “exclusion zones”) around schools (Rahilly et al., 2024). These management zones only affect new takeaways and are not able to impact those currently in operation. The shape, size, geographic anchor point, and types of school to which these management zones apply are also variable. Moreover, “full management zones”, a sub-type of takeaway management zone, prohibit all new takeaways, whereas “time management zones” only place limits on the opening hours of new takeaways. Some management zones exempt town centres where they overlap (“town centre exempt zones”), in an attempt to preserve the economic vitality of these areas.

It is anticipated that management zones could improve public health by reducing the number of new takeaways. Thereby minimising future exposure to takeaways and subsequently reducing unhealthy takeaway food consumption. While ostensibly targeting children (Keeble et al., 2021), it is anticipated that population-level takeaway exposure may also be reduced. In part this is because on average, 17% of land area within adopter LAs falls within a management zone (Rahilly et al., 2024). It is therefore likely that an even greater proportion of the population will be subject to this intervention, be that at home or through the course of their day-to-day activities.

Recent evidence indicates that adoption of takeaway management zones around schools from 2009 to 2017 by 35 LAs was associated with fewer new takeaway planning applications received, and more of these applications being rejected (Rahilly et al., 2024). Observed intervention effects varied by type of management zone adopted, with the impacts of full management zones most prominent. While this suggests that management zones may be able to reduce numbers of new takeaway outlets, this was not explicitly studied. Other research from a single LA did not find an effect on total numbers of takeaways (Brown et al., 2021). However, it is possible that this evaluation of a geographically specific intervention was underpowered to detect statistically significant effects or was confounded by local policy or other factors. Moreover, due to data availability, evaluations to date have reported outcomes measured over the short-term (<3 years). It is unknown whether any observed impacts associated with the intervention would be sustained in the longer term.

Management zones were originally designed to target class A5 hot food takeaways within the planning system in England (O’Malley et al., 2023). Other types of food retail such as cafes (class A1) and restaurants (class A3) are not subject to these regulations. For example, chain fast-food outlets such as McDonald's, Burger King and Kentucky Fried Chicken (KFC) are most often classified as restaurants (except, for example, those with a drive-thru facility) and are not subject to management zones regulations. However, chain fast-food outlets sell food of a similar nutritional profile, and often for consumption off the premises, as that sold in regulated A5 hot food takeaways (Robinson et al., 2018). One unintended effect of takeaway management zones could be the proliferation of chain fast-food outlets in their place. Another unintended impact of management zones could be displacement of new takeaways to the (unregulated) area immediately beyond their peripheries. Both of these possibilities could undermine the public health impacts of management zones, but neither have been formally studied.

These knowledge gaps are established barriers to further adoption and implementation of takeaway management zones (Keeble et al., 2021; O’Malley et al., 2021). This may explain why, despite a decade since first adoption, approximately 90% of LAs have still not adopted takeaway management zones around schools, despite endorsement and encouragement from national policy and planning guidance (Greater London Authority, 2012; Public Health England, 2018; Local Government Association, 2016).

In this study, we used existing data to study the number of new takeaways, before and after the adoption of takeaway management zones around schools, across all 26 adopter LAs in England from 2013 to 2017. This was a natural experimental evaluation using uncontrolled interrupted time series analyses, with results stratified by intervention sub-type.

## 2 Materials and methods

We used uncontrolled interrupted time series analyses to estimate changes from up to six years pre- and post-adoption of takeaway management zones around schools. We studied three outcomes: mean number of new takeaways within management zones (and by sub-type: full management, town centre exempt and time management zones); mean number of new takeaways on the periphery of management zones; and presence of new chain fast-food outlets within management zones.

### 2.1 Intervention LAs

**Fig. 1:**
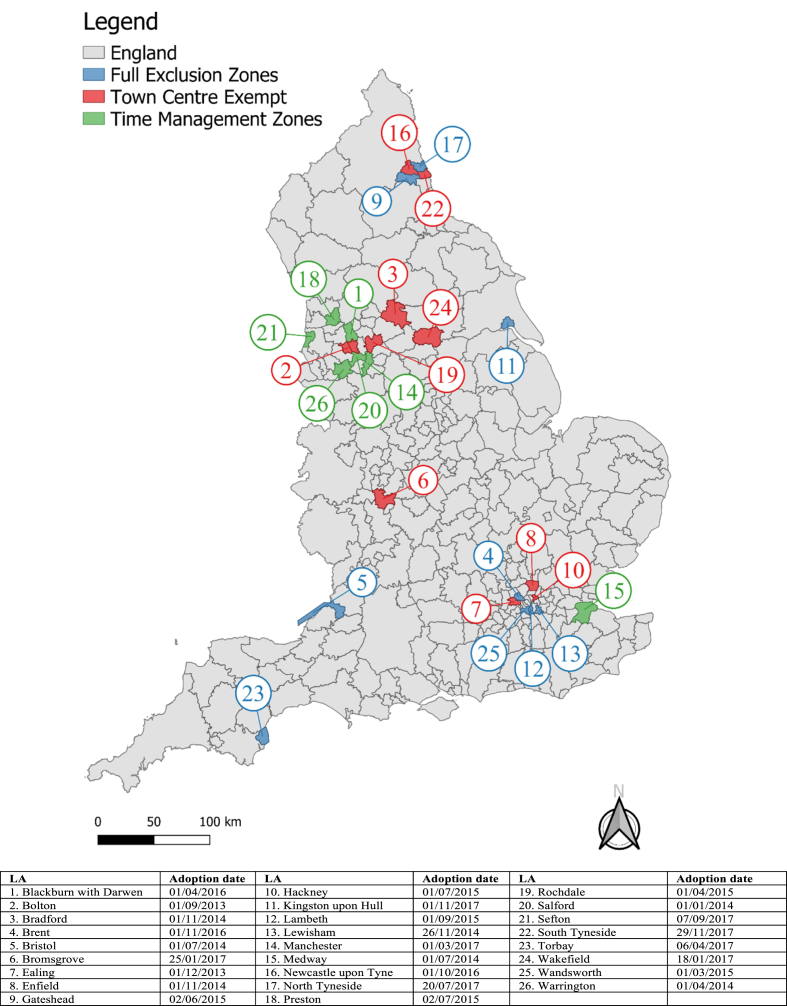
Local authorities (n = 26) that had adopted takeaway management zones (by type) around schools between September 1, 2013 and December 31, 2017.

We identified intervention LAs via freedom of information (FOI) requests, which were sent to all 325 LAs in England in June 2021. We found that since the first recorded adoption in Waltham Forest in 2009 (Keeble et al., 2019), 35 LAs in England had introduced takeaway management zones around schools by December 31, 2017 (Rahilly et al., 2024). Of these, 26% (9 of 35 LAs) adopted full management zones, 54% (19 of 35) adopted town centre exempt zones, and 20% (7 of 35) adopted time management zones. We analysed data for 26 LAs in England (Fig. 1) that adopted takeaway management zones around schools between September 2013 and December 2017. Earlier instances of adoption could not be evaluated due to lack of data availability, while analysis of later adopters risked being contaminated by temporary COVID-19 related planning amendments (Moore et al., 2022).

### 2.2 Management zones and peripheral areas

Takeaway management zones around schools were recreated using a geographic information system (PostGIS), according to specifications published by LAs or provided in response to FOI request. Briefly, using Edubase as our gold-standard database of schools, we overlaid polygon data from Ordnance Survey (OS) Mastermap Sites and Topography layers, to establish school boundaries and access points, and from which to calculate school centres. School boundaries, centres or access points were then buffered according to LA specifications in order to create management zones. Our approach to recreating management zones ensures that they are dynamic and responsive to new schools opening and others closing over time. For LAs that operate town centre exempt zones, we manually digitised town centre boundaries from static and interactive maps made available online by LAs. In addition, we created a 100 m ‘periphery’ around (i.e. on the outside edge of) these management zones, based on the presumption that this would be a high risk area if the intervention led to a displacement effect.

### 2.3 Food outlet data

We used food outlet data from OS Points of interest (POI) to identify newly opened: a) takeaways; and b) chain fast-food outlets. There is precedent for the use of OS POI data in research, as an accurate, historic and nationwide source of secondary data on the locations and types of food outlets in England (Wilkins et al., 2017). The data contains takeaway and chain fast-food outlets within its classification scheme (‘fast food and takeaway outlets’ (01020018), ‘fish and chip shops’ (01020020), and ‘fast food delivery services’ (01020019)). However, chain fast-food outlets are not readily distinguishable from takeaways. Moreover, combining these three classes does not equate to class A5 use. For example, the ‘fast food and takeaway outlet’ class includes sandwich shops that are not subject to regulation. Sandwich, ice cream and dessert shops were therefore removed using string identifiers present in the name field (see Supplementary Material B). Chain fast-food outlets were extracted by string matching according to a list of chain fast-food retailers provided by Public Health England (now the Office for Health Improvement and Disparities). Matched strings for chain fast-food outlets from the restaurant class (01020043) were also extracted and combined.

**Fig. 2:**
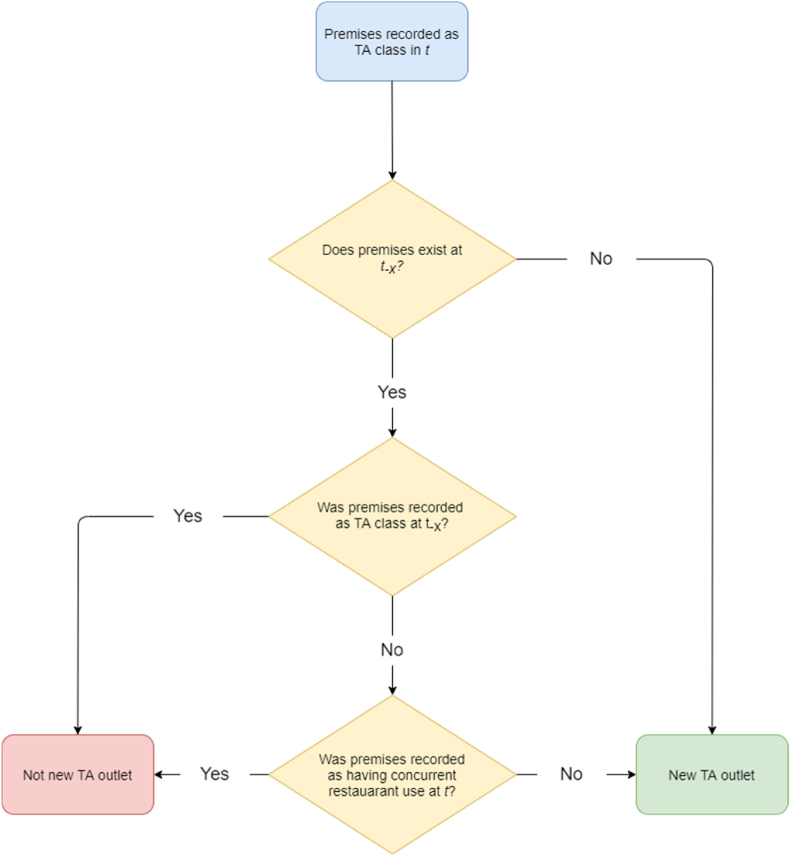
Schematic diagram of “new” takeaway outlet identification. New premises were identified based upon the unique reference, name and classification having not previously existed. Subsequent checks were made against topographic ID, address, street name and postcode. Throughout *t*_*-x*_ refers to the previous time point (quarter) at which the premises was recorded in OS POI data.

Quarterly, historic POI data were available directly from OS under an educational licence (Supplementary material A). This enabled us to construct a time series from June 1, 2011 to March 1, 2020. Data were available for consecutive quarters except in three instances: September 1, 2011, December 1, 2013, and March 1, 2014. The data contained a unique reference, topographic ID, name, address, street name, postcode, classification, date and location (with a stated 1 m precision) for each food outlet. Outlets were mapped, and within management zones a “new” outlet was initially identified as any premises at which the unique reference, name and classification did not exist at the prior time point. Topographic ID, address, street name and postcode were then used to confirm whether this outlet represented a genuinely new outlet or a change of use within existing premises, which would be subject to the intervention (Fig. 2). Where the time interval between two data points spanned a missing quarter, the number of new outlets was divided by the number of intervening quarters and therefore reflects the mean number of new outlets per quarter.

### 2.4 Data aggregation

To make the most of the available data, we calculated the mean number of new outlets per LA. Time was synchronised around the adoption date (*t*) for each local authority, with quarters wholly prior to the date of adoption defined as pre-intervention (*t*_*-n*_, …, *t*_*-1*_), and quarters wholly after the date of adoption defined as post-intervention (*t* + _*1*_, …,*t* + _*n*_). Due to data availability, the samples contributing to the mean at the extremes of the research periods were smaller than for the quarters closer to the date of intervention (Supplementary Material C1). To account for this, models were frequency weighted based on the number of LAs contributing data at each time point. Where an adoption date did not fall on the first day of a quarter, the relevant quarter was excluded to ensure clear separation between pre- and post-intervention periods. For example, where the adoption date was January 18, 2017, the number of new outlets that opened at quarter one of 2017 (beginning March 1, 2017) was not included as some of this quarter was under management zone restriction and some was not (Supplementary Material C2).

### 2.5 Outcomes

The primary outcome was:

1. Mean number of new takeaways within management zones around schools per LA per quarter.

Analyses of the primary outcome were also stratified by intervention sub-type: full management (n = 9 LAs), town centre exempt (n = 10 LAs) and time management zones (n = 7 LAs).

The secondary outcomes were:

2. Mean number of new takeaways on the periphery (within an additional 100m) of management zones around schools per LA per quarter.
3. Presence of any new chain fast-food outlet within management zones around schools per LA per quarter.

### 2.6 Statistical analysis

We used uncontrolled interrupted time series analyses, undertaken as segmented regression models, to estimate an intervention effect representing the difference between a modelled trend fitted to observed post-intervention data, and a counterfactual extrapolated from the pre-intervention trend. Results are reported as both level and trend changes. In addition, where level changes were statistically significant, differences are also shown between the point estimate and corresponding counterfactual at 12, 24, and 48 months, and at the maximum time interval post-intervention (66–72 months depending on the analytic sample). In addition we also report an estimated cumulative difference across the maximum extent of the post-intervention period.

Prior to analysis, simple linear regressions were modelled against the data. This facilitated Durbin-Watson (Turner et al., 2021) and Ljung-Box tests (Thayer et al., 2021), in conjunction with visual examination of auto-correlation plots, which suggested that the data were not auto-correlated. A Webel and Ollech test (Ollech & Webel, 2022) further confirmed the absence of seasonal patterns affecting the data. Pre-analytical checks were undertaken to determine optimal model type (options were OLS, ARIMA, GLM, GLS), with results compared using Root Mean Squared Errors (RMSEs). Consequently, OLS models (frequency weighted by sample size at each time point) were adopted for all primary analyses, as well as the number of new takeaways located on the periphery. Due to a high number of zero counts in the majority of management zones, we analysed only the presence of at least one new chain fast-food outlet, not the absolute number, using logistic regression (full model outline in Supplementary Material D). All final models were also checked for over-dispersion and autocorrelation (Supplementary Material E), which were not observed.

Data were analysed using *R* (*emmeans* and *margins* packages) and we applied a two-tailed significance α of 0.05.

### 2.7 Sensitivity analysis

A common approach designed to test whether any observed changes were specific to the time of intervention is known as “temporal falsification” (Craig et al., 2017). A separate analysis was run using data from the pre-intervention period only (22 quarterly observations), with an intervention at the midway point (t_-11_). If any observed changes from primary analyses were robust to the date of intervention proper, we would not expect to observe comparable changes at other times.

Additionally, where analyses were undertaken in regards to the number of new takeaways on the periphery of management zones, and presence/absence of new chain outlets within management zones, results were derived separately for each of the regulation sub-types.

## 3 Results

The majority (92%) of LAs adopting takeaway management zones around schools used a 400 m buffer. This buffer was applied to the boundary of the school site in 50% of LAs (n = 13), originated from the centre of schools in 19% of LAs (n = 5), and originated from school access points in 31% of LAs (n = 8).

### 3.1 Change in mean number of new takeaways within management zones

**Fig. 3:**
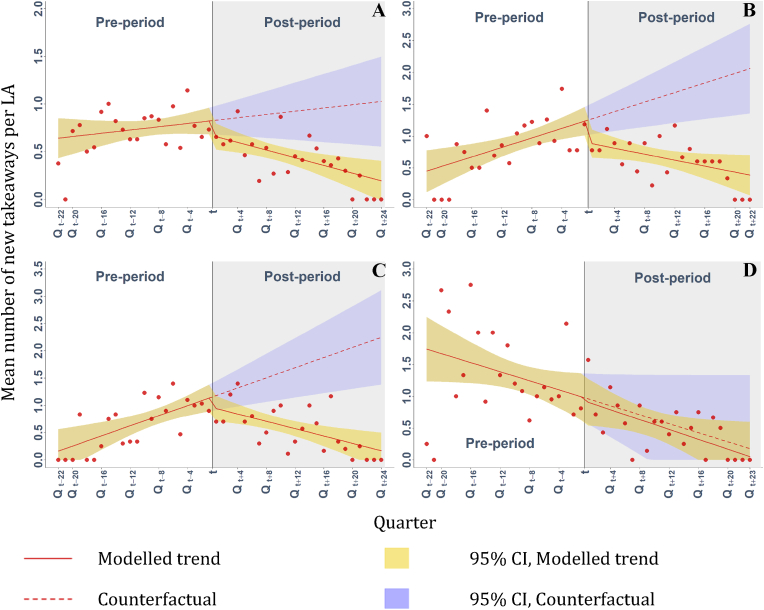
Mean number of new takeaways opening per quarter per local authority within takeaway management zones around schools, overall (A) and by regulation type; full management zones (B); town centre exempt zones (C); and time management zones (D). Modelled using uncontrolled interrupted time series analyses. Points are observed data. The vertical line represents when planning measures were adopted (t), defining pre- and post-intervention periods.

Overall, following the adoption of takeaway management zones around schools, the pre-intervention upward growth in mean number of new outlets per quarter per LA was reversed (Fig. 3A). During the pre-intervention period an increasing trend of 0.01 mean new outlets per quarter per LA was estimated. However, after the adoption of management zone restrictions this rate was reversed to a 0.02 decrease, reflecting a significant post-intervention trend change of −0.03 (95% CI -0.01, −0.05). Significant trend changes were also observed for both full management zones (Fig. 3B), at a rate of 0.06 (95% CI -0.03, −0.09) fewer new outlets per quarter per LA, and town centre exempt zones (Fig. 3C), at a rate of 0.08 (95% CI -0.04, −0.12) fewer new outlets per quarter per LA. However, adoption of time management zones (Fig. 3D) was associated with no significant change in post-intervention trend trajectory. For all outcomes, no statistically significant level changes were observed at the point of intervention.

Overall, driven by this significant trend reversal in the post-intervention period, adoption of management zones was associated with 0.26 (95% CI -0.03, −0.48; 30.3% fewer, 95% –CI 4.0, −56.6), 0.37 (95% CI -0.11, −0.64; 42.0% fewer, 95% CI -12.1, −71.9), 0.60 (95% CI -0.21, −0.96; 62.9% fewer, 95% CI -22.4, −100) and 0.83 (95% CI -0.30, −1.03; 81.0% fewer, 95% CI -29.1, −100) fewer new takeaways opening at 12, 24, 48 and 72 months respectively, than would have been expected in absence of the intervention (Table 1). Cumulatively over the entire post-intervention period, the result was 12 (54%) fewer new outlets opening within management zones than would have been otherwise anticipated..

**Table 1:** Estimated trend and level changes between pre- and post-intervention in terms of mean number of new takeaways opening within management zones around schools per quarter per LA within the sample, and differences at 12, 24 and 48 months, and at the maximum time interval (72 months for all management zones, 66 months for full management zones, 72 months for town centre exempt zones and 69 months for time management zones).

	β	95% CI	β	95% CI	β	95% CI	β	95% CI
	Management zones (n = 26)		Full management zones (n = 9)		Town centre exempt zones (n = 10)		Time management zones (n = 7)	
Pre-intervention Intercept (β_0_)	0.63**	0.40, 0.86	0.41*	0.05, 0.77	0.11	−0.32, 0.55	1.78**	1.22, 2.33
Pre-intervention Trend (β_1_)	0.01	−0.01, 0.02	0.04**	0.01, 0.06	0.05**	0.02, 0.07	−0.04	−0.07, 0.00
Post-intervention Level Change (β_2_)	−0.14	−0.35, 0.07	−0.33	−0.67, 0.07	−0.16	−0.52, 0.20	−0.05	−0.61, 0.51
Post-intervention Trend Change (β_3_)	−0.03**	−0.01, −0.05	−0.06**	−0.03, −0.09	−0.08**	−0.04, −0.12,	<0.01	−0.06, 0.05
Difference: 12 months	−0.26*	−0.03, −0.48,	−0.57**	−0.22, −0.92	−0.48**	−0.08, −0.88	−0.06	−0.65, 0.52
% Difference	−30.3	−4.0, −56.6	−41.5	−15.6, −67.4	−36.2	−6.2, −66.3	−7.5	−76.3, 61.3
Difference: 24 months	−0.37**	−0.11, −0.64	−0.82**	−0.40, −1.24,	−0.80**	−0.32, −1.27	−0.08	−0.76, 0.61
% Difference	−42.0	−12.1, −71.9,	−53.4	−25.8, −80.9	−53.1	−21.5, −84.6	−10.9	−100, 85.4
Difference: 48 months	−0.60**	−0.21, −0.96	−1.31**	−0.69, −1.84	−1.44**	−0.74, −2.14	−0.10	−0.43, 0.89
% Difference	−62.9	−22.4, −100	−71.3	−37.5, −100	−76.7	−39.5, −100	−24.2	−100, 208
Difference: Max months	−0.83**	−0.30, −1.03	−1.67**	−0.88, −2.06	−2.08**	−1.13, −3.04	−0.13	−0.18, 1.19
% Difference	−81.0	−29.1, −100	−81.3	−42.7, −100,	−92.6	−50.1, −100	−70.4	−100, 667

**p < 0.01, *p < 0.05; LA = local authority.

For full management zones, the observed reversal of trend from increasing to decreasing was associated with 0.57 (95% CI -0.22, −0.92; 41.5% fewer, 95% CI -15.6, −67.4), 0.82 (95% CI -0.40, −1.24; 53.4% fewer, 95% CI -25.8, −80.9), 1.31 (95% CI -0.69, −1.84; 71.3% fewer, 95% CI -37.5, −100) and 1.67 (95% CI -0.88, −2.06; 81.3% fewer, 95% CI -42.7, −100) fewer new takeaways opening at 12, 24, 48 and 66 months respectively, than would have been expected in absence of the intervention (Table 1). Over the entire post-intervention period, the result was 23 (62%) fewer new outlets opening within management zones than would have been otherwise anticipated.

For town centre exempt zones, the observed reversal of trend from increasing to decreasing was associated with 0.48 (95% CI -0.08, −0.88; 36.2% fewer, 95% CI -6.2, −66.3), 0.80 (95% CI -0.32, −1.27; 53.1% fewer, 95% CI -21.5, −84.6), 1.44 (95% CI -0.74, −2.14; 76.7% fewer, 95% CI -39.5, −100) and 2.08 (95% CI -1.13, −3.04; 92.6% fewer, 95% CI -50.1, −100) fewer new takeaways opening at 12, 24, 48 and 72 months respectively, than would have been expected in absence of the intervention (Table 1). Over the entire post-intervention period, the result was 28 (68%) fewer new outlets opening within management zones than would have been otherwise anticipated.

Neither level nor trend changes were associated with the adoption of time management zone restrictions. Differences at 12, 24, 48 and 69 months were also not statistically significant (Table 1).

### 3.2 Change in mean number of new takeaways in management zone periphery

**Fig. 4:**
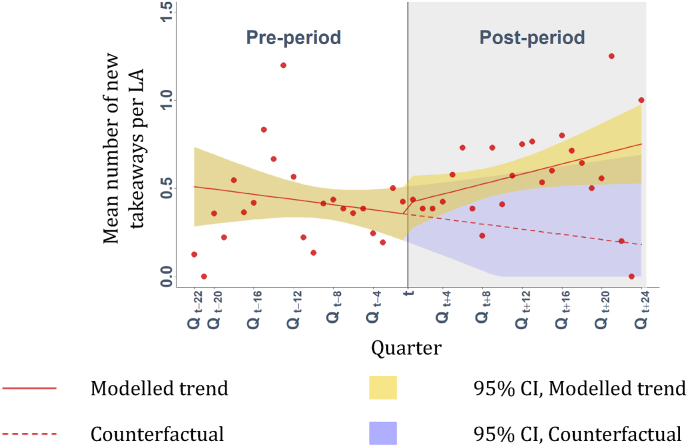
Mean number of new takeaways opening per quarter per local authority on the periphery of takeaway management zones around schools. Modelled using uncontrolled interrupted time series analyses. Points are observed data. The vertical line represents when planning measures were adopted (t), defining pre- and post-intervention periods.

There were no statistically significant trend or level changes observed (Fig. 4) in the mean number of new outlets per quarter per LA on the periphery of management zones, following their adoption (Table 2).

**Table 2:** Estimated trend and level changes between pre- and post-intervention in terms of mean number of new takeaways opening on the periphery of management zones around schools per quarter per LA (n = 26).

	β	95% CI
Management zone periphery (n = 26 LAs)		
Pre-intervention Intercept (β_0_)	0.52*	0.27, 0.76
Pre-intervention Trend (β_1_)	−0.01	−0.02, 0.01
Post-intervention Level Change (β_2_)	0.05	−0.17, 0.28
Post-intervention Trend Change (β_3_)	0.02	0.00, 0.04
Difference: 12 months	0.14	−0.10, 0.38
% Difference	42.9	−31.4, 117
Difference: 24 months	0.23	−0.06, 0.51
% Difference	76.1	−20.2, 173
Difference: 48 months	0.40	−0.02, 0.82
% Difference	167	−8.1, 342
Difference: Max months	0.57	<0.01, 1.15
% Difference	317	−1.6, 635

**p < 0.01; LA = local authority.

### 3.3 Change in the presence of new chain fast-food outlets within management zones

**Fig. 5:**
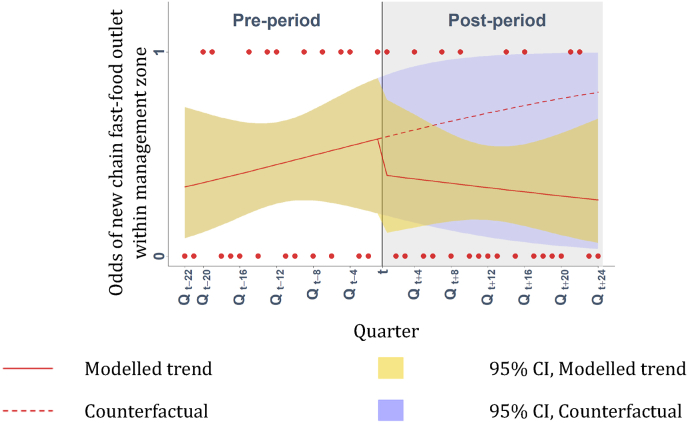
Difference in the odds of a new chain fast-food outlet having opened within takeaway management zones around schools per quarter per local authority. Modelled using uncontrolled interrupted time series analyses. Points are observed data. The vertical line represents when planning measures were adopted (t), defining pre- and post-intervention periods.

Following the adoption of takeaway management zones around schools, no statistically significant trend or level changes were observed (Fig. 5) in the odds of any new chain fast-food outlet having opened within management zones (Table 3).

**Table 3:** Estimated trend and level changes between pre- and post-intervention in terms of the odds of any new chain fast-food outlet having opened within management zones around schools (n = 26).

	OR	95 % CI
Management zones (n = 26)		
Pre-intervention Intercept (β_0_)	0.48	0.07, 2.81
Pre-intervention Trend (β_1_)	1.05	0.92, 1.21
Post-intervention Level Change (β_2_)	0.50	0.04, 5.25
Post-intervention Trend Change (β_3_)	0.93	0.77, 1.11
Difference: 12 months	0.38	0.03, 4.51
Difference: 24 months	0.28	0.02, 4.65
Difference: 48 months	0.16	0.01, 7.11
Difference: 72 months	0.09	0.01, 13.3

### 3.4 Sensitivity analysis

After synthesising an intervention at the mid-point of the pre-intervention period, we did not observe any statistically significant changes pre to post (Supplementary Material F), indicating that our findings are robust to the date of intervention.

Neither statistically significant level or trend changes in mean number of new takeaways on the periphery of management zones were observed for any analyses by regulation sub-type. Similarly, based upon the presence/absence of new chain fast-food outlets within management zones, no level or trend effect was estimated by regulation sub-type (Supplementary Material G).

## 4 Discussion

This is the first nationwide study of the retail impacts of takeaway management zones (sometimes referred to by LAs as “exclusion zones”) around schools. In all 26 LAs that adopted from 2013 to 2017, we observed an overall decrease in the number of new takeaways opening within management zones. At six years post-intervention, we observed 0.83 (81%) fewer new outlets opening per LA than would have been otherwise expected. Cumulatively over six years, 12 (54%) fewer new takeaways opened than would have been expected, driven primarily by divergent pre- and post-intervention trends. When stratified by policy type, effects were most prominent for full management zones and town centre exempt zones. Post-intervention, we observed 1.31 (71%) and 1.44 (77%) fewer new outlets opening, respectively, per LA at four years, and more exaggerated effects after this time horizon up to six years post-intervention. These changes equated cumulatively to 23 (62%) fewer new takeaways having opened over a 66 month post-intervention period among LAs adopting full management zones, and 28 (68%) fewer having opened among those excluding town centres over 72 months. Adoption of time management zones was not associated with a significant change in the number of new outlets at any post-intervention time point nor overall. There was no statistically significant evidence that the intervention had an effect on numbers of new takeaways opening on the periphery (i.e. within an additional 100 m) of management zones, nor on the opening of new chain fast-food outlets within management zones.

Our findings suggest that management zones have been effective in reversing a pre-intervention increasing trend in the number of new takeaways opening within close proximity to schools. This is consistent with previous work in which we showed that management zones were associated with a decrease in the number of planning applications received by LAs for takeaways, and an increase in the proportion of these applications that were rejected (Rahilly et al., 2024). Previous national and local analyses were limited to observations over relatively short two- or three-year post-intervention periods (Brown et al., 2021, 2022; Rahilly et al., 2024). In this analysis we were able to observe changes up to six years post-intervention. Over this period the impact of the intervention appeared to grow, complementing previous observations of a greater (albeit insignificant) effect of management zones on planning applications at 24 vs 12 months post-intervention (Rahilly et al., 2024). We hypothesise this is due to increased awareness of the regulations among prospective takeaway owners over time. Consequent to the impacts on takeaway retail we describe here, we would expect a reduction in population exposure to takeaways over this six-year term, relative to a scenario where no intervention was adopted. While there are many other factors that contribute to dietary consumption, management zones therefore have the potential to improve population health. It is also plausible, although not empirically explored here, that in conjunction with takeaway closures (unrelated to intervention), management zones could ultimately lead to a reduction in the *total* number of takeaways.

Historically, planning inspectors have cited a lack of evidence regarding the effectiveness of management zones for curbing the proliferation of takeaways as a material consideration in their decision-making, including during the London Plan review of 2019. Elsewhere, a general lack of research evidence in this space has been documented as curtailing the ability of LAs to adopt and effectively implement population-health focussed urban planning interventions addressing takeaways (Keeble et al., 2021; Nixon et al., 2015; O’Malley et al., 2021). However, while it is understandable that LAs are hesitant to waste their limited resources on interventions that have not been proved effective, our evidence means this is increasingly not the case for takeaway management zones around schools. Our results may also be internationally applicable to settings with similar regulatory levers in urban planning, such as in Australia and the US. These are contexts in which other forms of planning intervention to address unhealthy food retail have also so far been deemed to have failed (Sturm & Cohen, 2009; Sturm & Hattori, 2015).

When stratified by policy type, intervention effects were most prominent among LAs who adopted full management zones and town centre exempt zones. The former reflects earlier findings that full management zones were associated with an increase in the proportion of applications rejected by LAs at 12 (38.6% more, p < 0.05) and 24 (46.1% more, p < 0.05) months post-intervention. These consistent findings are evidence of an intervention effect associated with the adoption of management zones that include areas identified as town centres. However, in contrast to our findings here, previous observations of the effects of town centre exempt zones on more proximal outcomes were null, in both a nationwide study (Rahilly et al., 2024), and within an individual LA (Brown et al., 2021). It is possible that this apparent discrepancy is attributable to the extended post-intervention period of this study. Primarily driven by an inverted trend change, intervention effects here became more marked over time (up to six years post-intervention), whereas prior analyses have been restricted to two- or three-year post-intervention periods, which may have been insufficient to observe these effects.

Notably, and as previously, time management zones were not observed to reduce numbers of new takeaways in this analysis (Rahilly et al., 2024). We take this as further evidence that regulating hours of operation alone does not serve as a sufficient deterrent to new takeaways opening on the school fringe. This does not preclude the possibility that time management zones influence the consumption of takeaway food by children and young people through restricting temporal (rather than geographical) access to takeaway food. Further research is necessary to explore this.

While not significant (p = 0.06), we did observe weak evidence of an overall increase in the number of new takeaways on the periphery of management zones. Further, it is possible that the limited spatial extent that we operationalised for these peripheral areas, which captured relatively few new takeaways over even a six-year period, was responsible for our inability to detect statistical significance. Our weak evidence could therefore be interpreted as indicative of displacement, with new takeaways adapting their location practices in response to zone regulations. This is a potential public health concern. Further research is needed to understand this potential unintended impact, including how displacement could also shift the overall geography of takeaway retail within LAs and affect takeaway exposure in whole populations.

Our results suggest that adoption of takeaway management zones around schools was not associated with numbers of new chain fast-food outlets up to six years post-intervention. Therefore it would seem that new chain fast-food outlets are not meeting any residual demand for takeaway-type food (at least within management zones). From a public health perspective, this appears to be a strength of this intervention. However, numbers of chain fast-food outlets continue to increase over time in the UK and elsewhere (Statista, 2023). McDonald's has published its plans to expand in out-of-town retail and on major arterial road locations, where premises can be larger (minimum 3600 sq ft) and are more accessible at least by car (McDonald's, 2023). These locations may be less likely to fall within close proximity to schools, hence our observation that their numbers in these areas did not increase. Within management zones, further longitudinal research is required at the retail unit level to understand which retail uses do take the place of takeaways, including whether or not these new uses have public health impacts.

### 4.1 Strengths and limitations

Through using mean number of new outlets per LA, accounting for different sample sizes at each time point through frequency weighting, a strength of our analysis was the ability to evaluate this intervention over a period of up to six years post-adoption, thereby increasing our ability to detect significant effects. Previous observational analyses of management zone interventions have been restricted to a post-intervention period of three or fewer years (Brown et al., 2021, 2022; Rahilly et al., 2024), in which it was potentially less likely that impacts would be observable. This would be particularly so for the more distal outcome of takeaway retail studied here.

All analyses were uncontrolled and may therefore be subject to confounding from unmeasured, coincident events (Hategeka et al., 2020). However, we took a number of steps to mitigate against this. First, we synchronised time such that intervention time-point *t* represented 26 individual adoption dates ranging from September 2013 to December 2017. Confounding from coincident national-level events is therefore considered unlikely. Second, our nationwide coverage minimises the possibility of confounding from locally-specific coincident events. Third, sensitivity analyses revealed that the effects observed were specific to, at least, the time of intervention.

We used data from OS POI, which is an accurate, historic database of food outlet locations across England (Burgoine & Harrison, 2013; Wilkins et al., 2017). There is precedent for the use of OS POI data in previous food environment research (Hobbs et al., 2019). We also re-classified OS POI data in order to delineate class A5 hot food takeaway and chain fast-food outlets, and reduce error resulting from any pre-existing misclassification. However, as these amendments relied partly on automated string matching, it is likely that some outlets may still have been misclassified. For example, a dessert or sandwich shop that was erroneously listed in POI data as a takeaway, but whose name did not contain a match against a list of key strings (Supplementary Material B), would not have been removed from the takeaway class. Moreover, there were classification inconsistencies over time between POI data releases, even for the same outlet. We developed an algorithm to detect duplication of records based upon multiple data fields and minimise error. The management zones we recreated were based on specifications published by LAs, which also minimises potential for error.

We aggregated data for LAs, masking any potential heterogeneity in impacts across LAs adopting management zones. Instead, our analyses offer a broad insight into the typical effect of such interventions. Moreover, while these impacts may be generalizable to other similar LAs (those included in this study were predominantly urban and relatively more deprived, of which there are many more in England), they may not be as generalizable to all. Further work could examine the potential for differing intervention impacts across different types of LAs. However, even in rural LAs, locations of schools and takeaways are likely to collocate around pockets of urban development, which may offer the possibility that the intervention could also be effective in this type of LA.

## 5 Conclusions

This is the first nationwide study of the retail impacts of takeaway management zones around schools (sometimes referred to by LAs as “exclusion zones”). In all 26 LAs that adopted from 2013 to 2017, we observed an overall decrease in the number of new takeaways opening within management zones. At six years post-intervention, we observed 0.83 (81%) fewer new outlets opening per LA than would have been otherwise expected in absence of the intervention. Cumulatively, 12 (54%) fewer new takeaways opened than would have been expected over a six year post-intervention period. When stratified by policy type, effects were most prominent for full management zones and town centre exempt zones. Estimates of intervention effects on numbers of new takeaways on the periphery (i.e. within an additional 100 m) of management zones, and on the presence of new chain fast-food outlets within management zones, did not meet statistical significance. Our findings suggest that management zone policies were able to demonstrably curb the proliferation of new takeaways. Modelling studies are required to measure the possible population health impacts associated with this change.

## Ethics approval and consent to participate

Not applicable.

## Consent for publication

Not applicable.

## Availability of data and materials

This study used third party data made available under licence from OS that the author does not have permission to share. Requests to access the data should be directed to OS.

## Funding

This study is funded by the 10.13039/501100000272National Institute for Health Research (10.13039/501100000272NIHR) 10.13039/501100001921Public Health Research Programme (Project number: NIHR130597). The views expressed are those of the author(s) and not necessarily those of the NIHR or the Department of Health and Social Care. JR, YH, MK, BL, AS, SJS, MW, JA and TB were supported by the 10.13039/501100000265Medical Research Council (grant number MC_UU_00006/7). OM is supported by a 10.13039/100014013UKRI Future Leaders Fellowship (MR/T041226/1). For the purpose of open access, the author has applied a Creative Commons Attribution (CC BY) licence to any Author Accepted Manuscript version arising.

## CRediT authorship contribution statement

**John Rahilly:** Writing – review & editing, Writing – original draft, Visualization, Methodology, Formal analysis, Data curation, Conceptualization. **Ben Amies-Cull:** Writing – review & editing. **Michael Chang:** Writing – review & editing, Methodology, Funding acquisition, Conceptualization. **Steven Cummins:** Writing – review & editing, Methodology, Funding acquisition, Conceptualization. **Daniel Derbyshire:** Writing – review & editing. **Suzan Hassan:** Writing – review & editing. **Yuru Huang:** Writing – review & editing, Data curation. **Matthew Keeble:** Writing – review & editing. **Bochu Liu:** Writing – review & editing. **Antonieta Medina-Lara:** Writing – review & editing. **Oliver Mytton:** Writing – review & editing, Methodology, Funding acquisition, Conceptualization. **Nina Rogers:** Writing – review & editing. **Bea Savory:** Writing – review & editing. **Annie Schiff:** Writing – review & editing, Data curation. **Stephen J. Sharp:** Writing – review & editing, Methodology, Funding acquisition, Conceptualization. **Richard Smith:** Writing – review & editing, Methodology, Funding acquisition, Conceptualization. **Claire Thompson:** Writing – review & editing, Methodology, Funding acquisition, Conceptualization. **Martin White:** Writing – review & editing, Methodology, Funding acquisition, Conceptualization. **Jean Adams:** Writing – review & editing, Methodology, Funding acquisition, Conceptualization. **Thomas Burgoine:** Writing – review & editing, Writing – original draft, Validation, Supervision, Methodology, Funding acquisition, Formal analysis, Data curation, Conceptualization.

## Declaration of competing interest

The authors declare that they have no competing interests.

## Data Availability

The authors do not have permission to share data.
