# Supplementary material for: Changes in the number of new takeaway food outlets associated with adoption of management zones around schools: A natural experimental evaluation in England
Source: SSM Popul Health. 2024 Mar 19;26:101646. doi: 10.1016/j.ssmph.2024.101646 (PMC11033196; doi:10.1016/j.ssmph.2024.101646)
Supplement: Multimedia component 1 [file mmc1.docx]

**Supplementary Material A:** OS POI data archive dates and quarterly time intervals between consecutive archive dates.

| **Archive Date** | 01/06/2011 | 01/12/2011 | 01/03/2012 | 01/06/2012 | 01/09/2012 | 01/12/2012 |
| --- | --- | --- | --- | --- | --- | --- |
| **Time Interval**  **(Quarters)** | *NA* | *2* | *1* | *1* | *1* | *1* |
| **Archive Date** | 01/03/2013 | 01/06/2013 | 01/09/2013 | 01/06/2014 | 01/09/2014 | 01/12/2014 |
| **Time Interval**  **(Quarters)** | *1* | *1* | *1* | *3* | *1* | *1* |
| **Archive Date** | 01/03/2015 | 01/06/2015 | 01/09/2015 | 01/12/2015 | 01/03/2016 | 01/06/2016 |
| **Time Interval**  **(Quarters)** | *1* | *1* | *1* | *1* | *1* | *1* |
| **Archive Date** | 01/09/2016 | 01/12/2016 | 01/03/2017 | 01/06/2017 | 01/09/2017 | 01/12/2017 |
| **Time Interval**  **(Quarters)** | *1* | *1* | *1* | *1* | *1* | *1* |
| **Archive Date** | 01/03/2018 | 01/06/2018 | 01/09/2018 | 01/12/2018 | 01/03/2019 | 01/06/2019 |
| **Time Interval**  **(Quarters)** | *1* | *1* | *1* | *1* | *1* | *1* |
| **Archive Date** | 01/09/2019 | 01/12/2019 | 01/03/2020 |  |  |  |
| **Time Interval**  **(Quarters)** | *1* | *1* | *1* |  |  |  |

**Supplementary Material B:** List of string terms identified as associated with ice-cream shops, sandwich shops and dessert shops.

| **String Variant** | **Amended classification** | **String Variant** | **Amended Classification** |
| --- | --- | --- | --- |
| Baguette | Other food outlet | Donut | Other food outlet |
| Bakery | Other food outlet | Gelato | Other food outlet |
| Baskin Robbins | Other food outlet | Ice | Other food outlet |
| Baskin Robins | Other food outlet | Krispy Kreme | Other food outlet |
| Cake | Other food outlet | Sandwich | Other food outlet |
| Candy | Other food outlet | Smoothie | Other food outlet |
| Cob | Other food outlet | Smoothies | Other food outlet |
| Cream | Other food outlet | Sweets | Other food outlet |
| Crepe | Other food outlet | Upper Crust | Other food outlet |
| Dessert | Other food outlet | Waffle | Other food outlet |
| Doughnut | Other food outlet | Waffles | Other food outlet |

Reclassification based upon the above string terms utilized a fuzzy matching approach in which all punctuation and capitalization was removed from relevant data columns (OS POI *name*).

**Supplementary Material C1:** Example of aggregation method based upon data from full sample (n = 26).

| **Time related to intervention (*t*)** | ***t_-22_*** | ***t­_-16_*** | ***t_-8_*** | ***t_-1_*** | ***t*** | ***t_+1_*** | ***t_+8_*** | ***t_+16_*** | ***t_+24_*** |
| --- | --- | --- | --- | --- | --- | --- | --- | --- | --- |
| Number of LAs in sample | 4 | 12 | 26 | 26 |  | 26 | 26 | 15 | 1 |
| New outlets per quarter | 2 | 11 | 22 | 19 |  | 17 | 14 | 6 | 0 |
| Mean new outlets per LA | 0.5 | 0.9 | 0.8 | 0.7 |  | 0.7 | 0.5 | 0.4 | 0 |

**Supplementary Material C2:** Example of coalesced time for three local authorities with different adoption dates (t). Time t-1 represents the end of the quarter immediately preceding the adoption date; t-8 the end of the quarter at eight time points prior to the adoption date; t+1 the end of the quarter immediately after the adoption date; and t+8 the end of the quarter eight time points after adoption.

| **Local Authority** | ***t_-8_*** | ***t_-1_*** | ***t*** | ***t_+1_*** | ***t_+8_*** |
| --- | --- | --- | --- | --- | --- |
| Bristol | 01/03/2012 | 01/06/2014 | 01/07/2014 | 01/12/2014 | 01/09/2016 |
| Wakefield | 01/03/2015 | 01/12/2016 | 18/01/2017 | 01/06/2017 | 01/03/2019 |
| South Tyneside | 01/12/2015 | 01/09/2017 | 29/11/2017 | 01/03/2018 | 01/03/2020 |

**Supplementary Material D:** Statistical Model for Interrupted Time Series analysis

$$Y_{\tau=}\beta_{0}+ \beta_{1}\tau+ \beta_{2}I+ \beta_{3}IT$$

Where *Yτ* represents the outcome variable at time τ; β_0_ represents the pre-intervention intercept; β_1_ the pre-intervention trend; τ a continuous variable reflecting the time since the start of the time period (1, …, *n*); β_2_ the estimated change in level at the point of intervention; *I* a dummy variable to differentiate between pre- and post-intervention periods (0,1); β_3_ the difference between the trend in the post-intervention period and the pre-intervention period; whilst *IT* is a continuous variable representing time (quarters) since intervention (0, 1,…, *n*).

**Supplementary Material E:** Autocorrelation plots based upon final ITS models

| 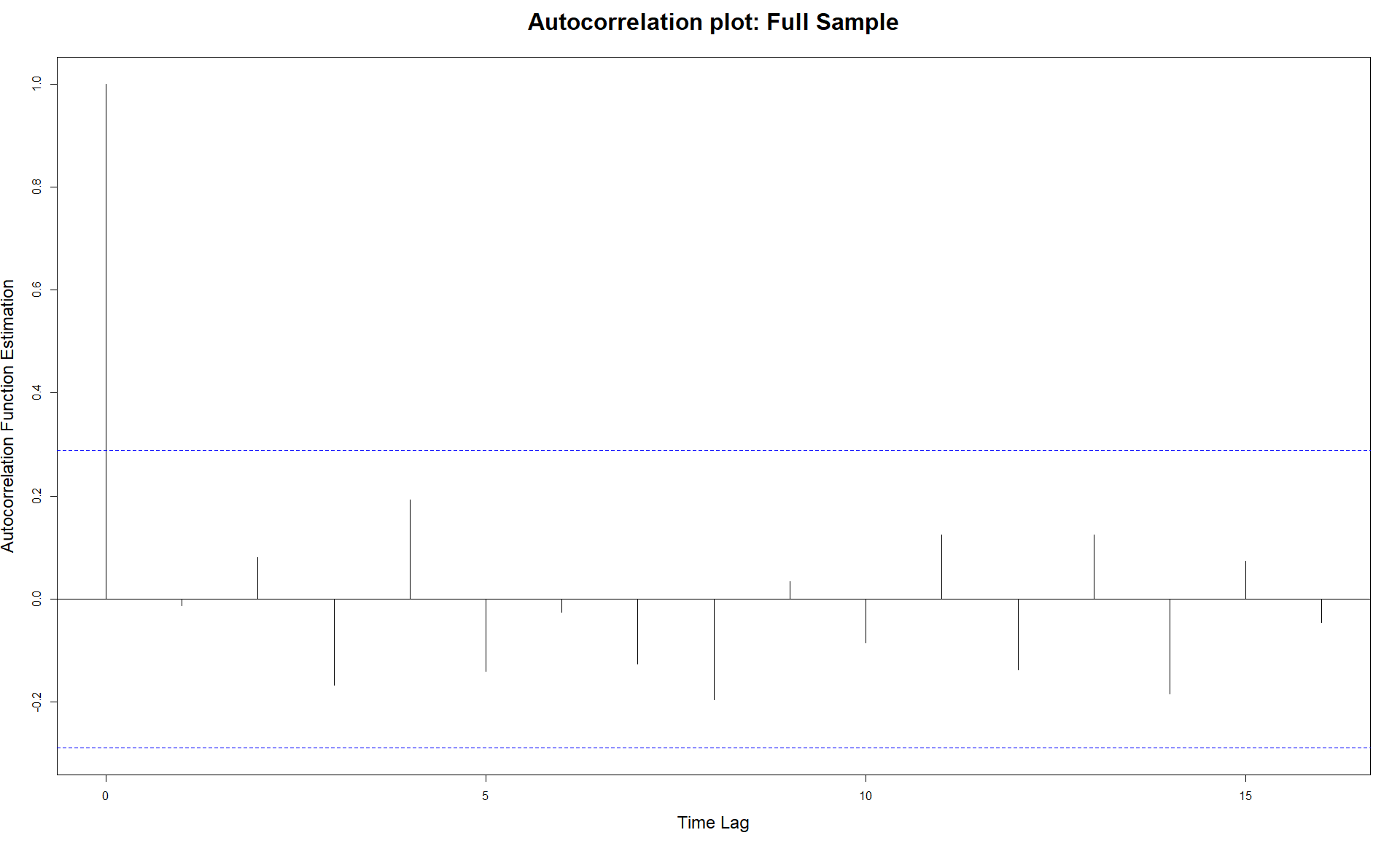 |
| --- |
| 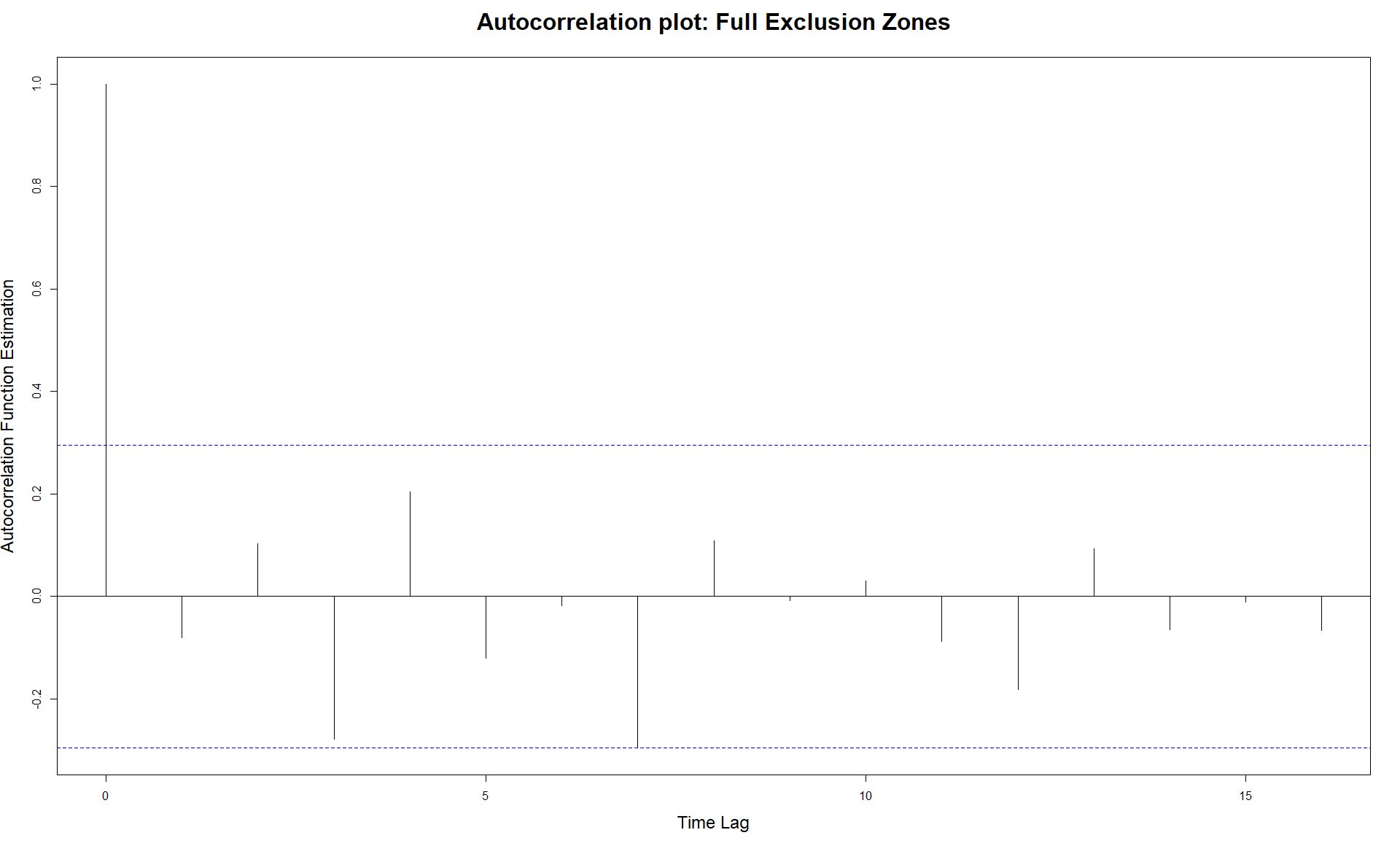 |
| 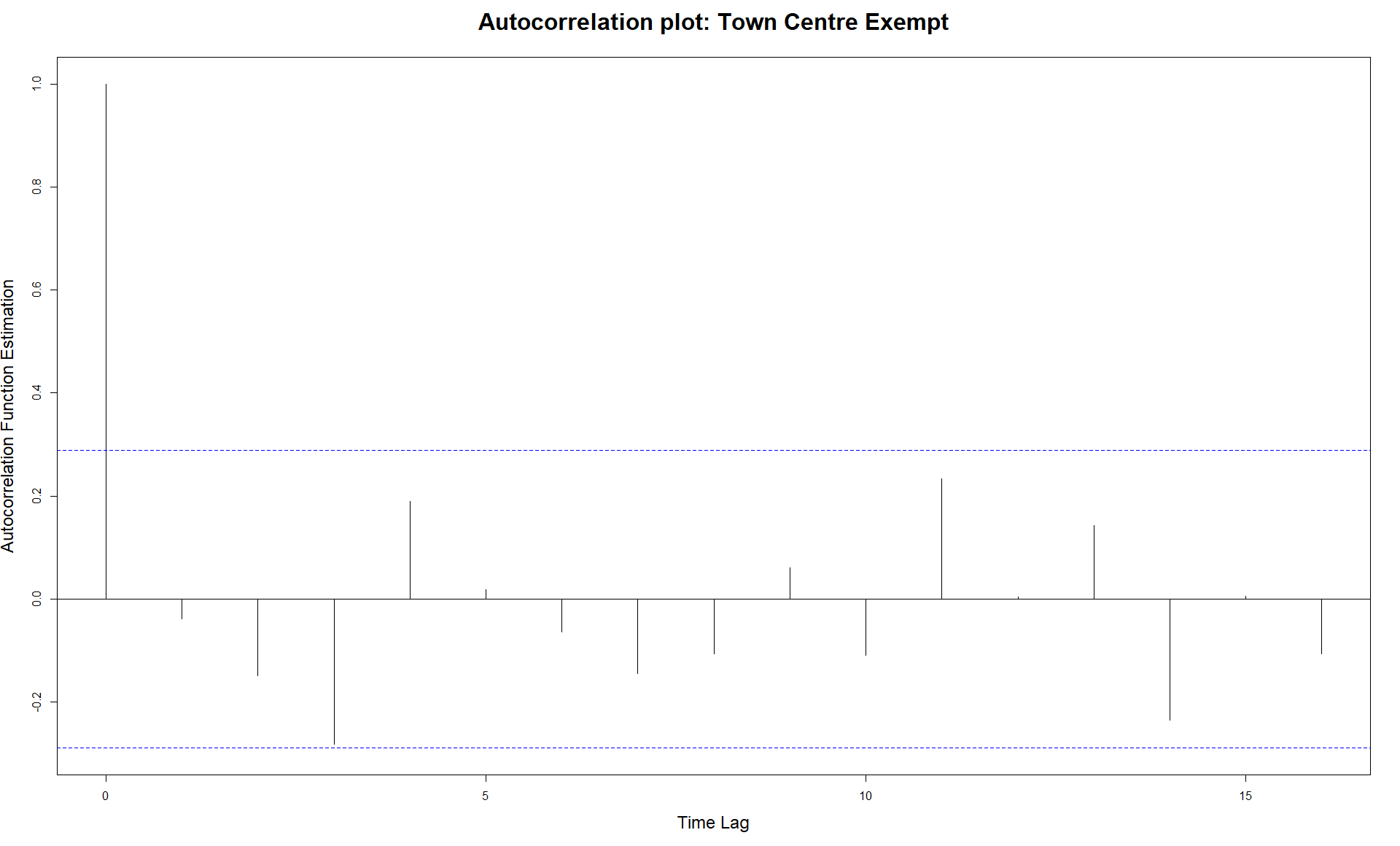 |
| 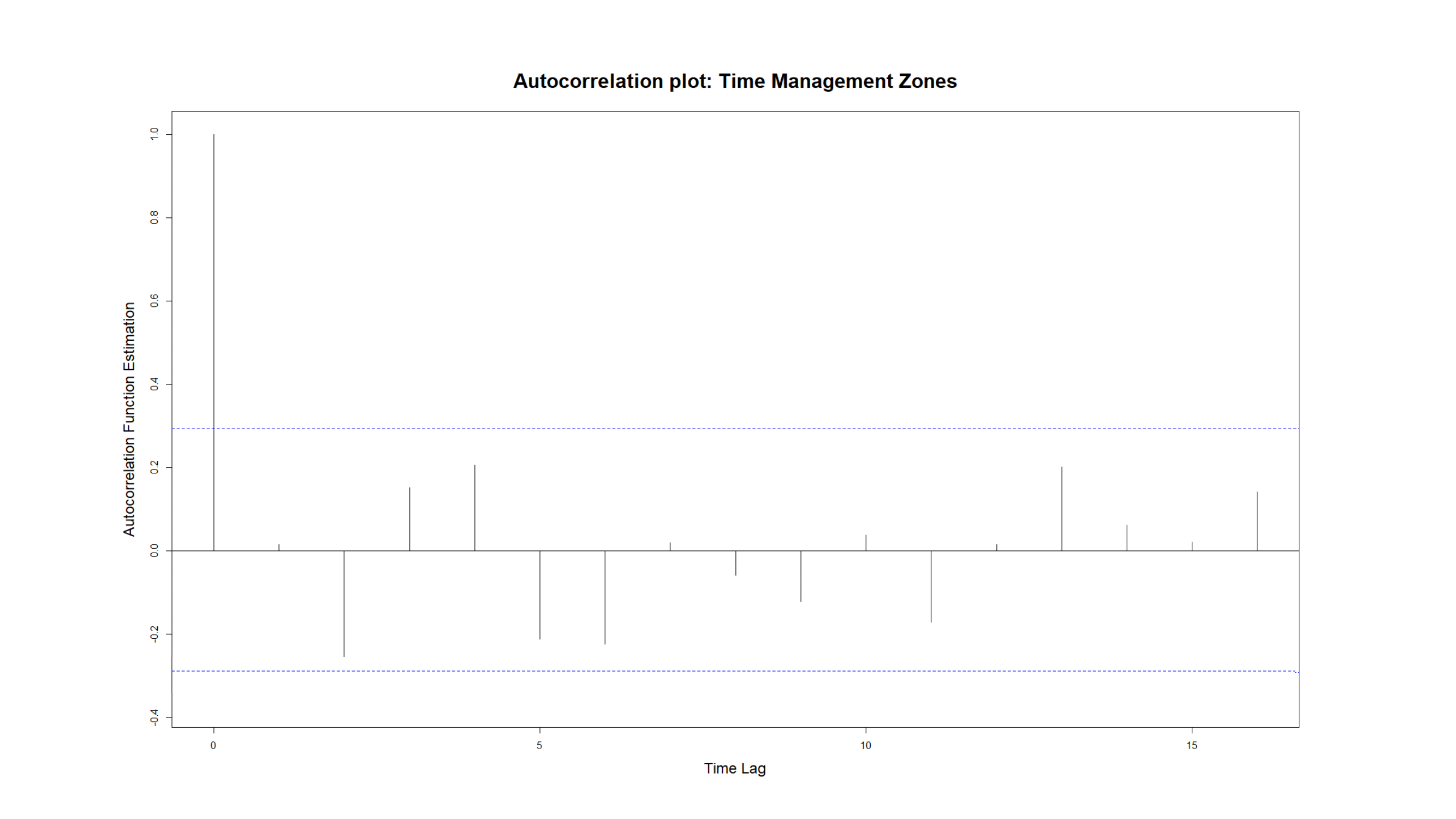 |
| 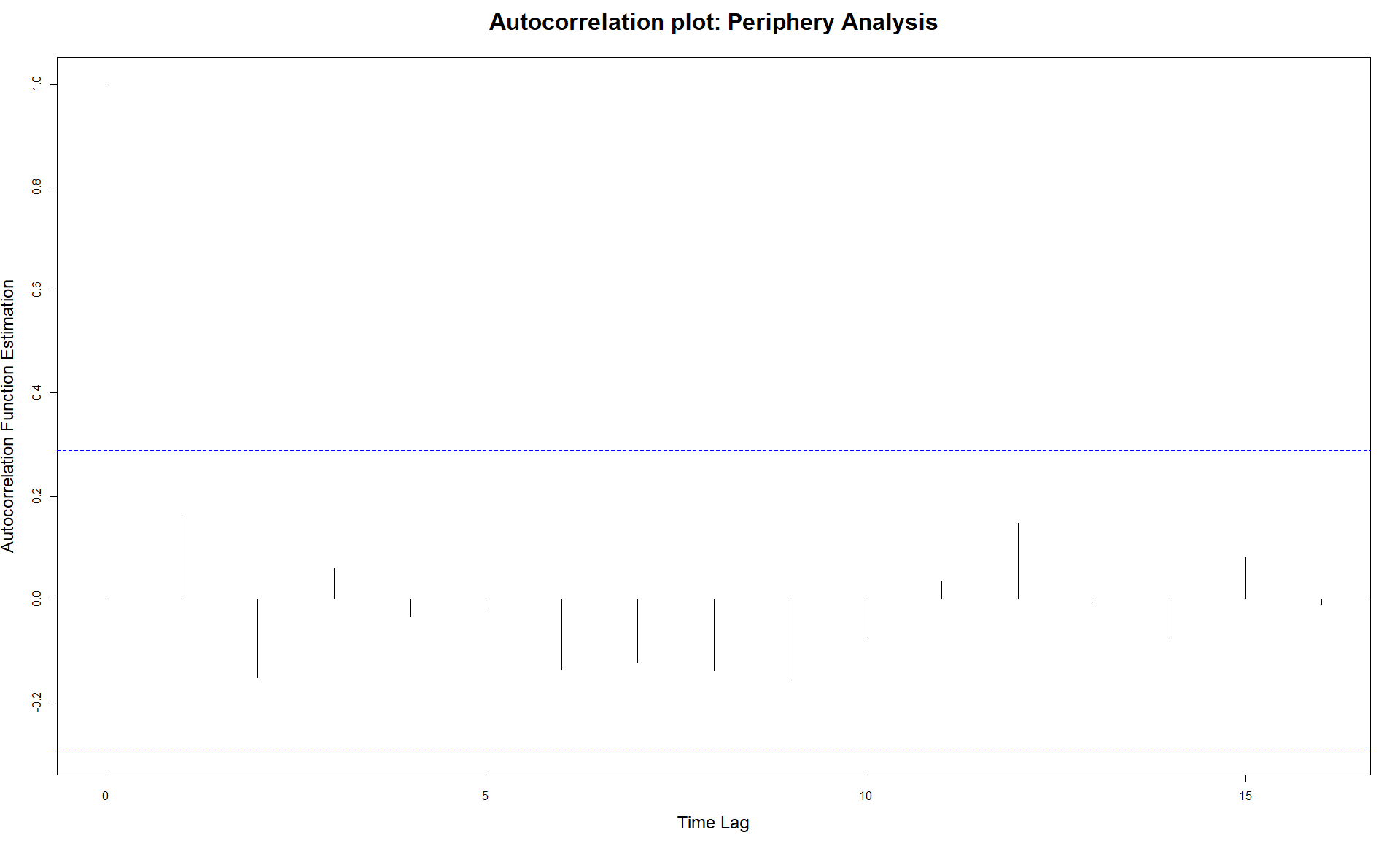 |
| 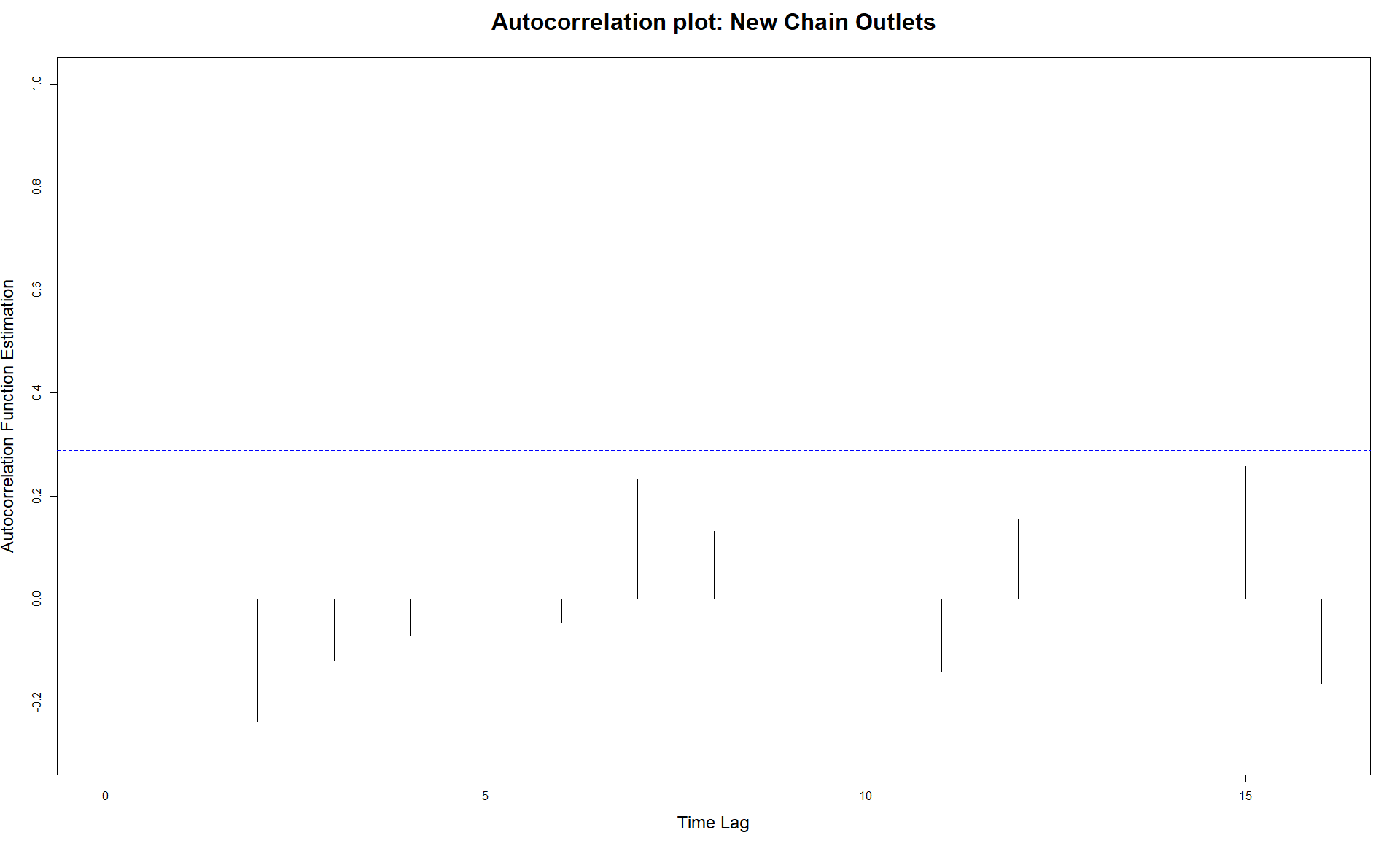 |

**Supplementary Material F:** This table presents the modelled outcomes for all analyses where the intervention date has been shifted to the mid-point within the pre-intervention period in order to test for the absence of an intervention effect. No statistically significant effects were estimated based upon any analyses.

|  | **Post-intervention Level Change** | | **Post-intervention Trend Change** | |
| --- | --- | --- | --- | --- |
|  | **β** | **P-value** | **β** | **P-value** |
| **Management zones**  **(n = 26)**  Original Analysis | -0.16 | 0.38 | -0.08 | 0.23 |
| Pre-intervention subset  (t = t_-11_) | -0.02 | 0.89 | -0.03 | 0.30 |
| **Full management zones**  **(n = 9)**  Original Analysis | -0.33 | 0.01* | -0.06 | 0.41 |
| Pre-intervention subset  (t = t_-11_) | 0.02 | 0.96 | -0.07 | 0.23 |
| **Town centre exempt zones**  **(n = 10)**  Original Analysis | -0.16 | 0.38 | -0.08 | 0.64 |
| Pre-intervention subset  (t = t_-11_) | 0.40 | 0.18 | -0.02 | 0.70 |
| **Time management zones**  **(n = 7)**  Original Analysis | -0.05 | 0.85 | <0.01 | 0.90 |
| Pre-intervention subset  (t = t_-11_) | -0.55 | 0.33 | -0.06 | 0.53 |

**Supplementary Material G:** This table presents the modelled outcomes for (A) number of new takeaways on the periphery of management zones, and (B) presence/absence of new chain outlets within management zones, based upon stratified analyses of regulation subtype. There were no statistically significant changes estimated across all analyses.

**A**

|  | **β** | **95% CI** | **β** | **95% CI** | **β** | **95% CI** |
| --- | --- | --- | --- | --- | --- | --- |
|  | **Full management zones**  **(*n* = 9)** | | **Town centre exempt zones**  **(*n* = 10)** | | **Time management zones**  **(*n* = 7)** | |
| Pre-intervention Intercept (β_0_) | 0.18 | -0.06, 0.42 | 0.80 | 0.34, 1.27 | 0.64 | 0.24, 1.03 |
| Pre-intervention Trend (β_1_) | 0.01 | -0.01, 0.02 | -0.02 | -0.05, 0.01 | -0.01 | -0.04, 0.01 |
| Post-intervention Level Change (β_2_) | -0.08 | -0.31, 0.14 | 0.24 | -0.14, 0.62 | 0.05 | -0.35, 0.45 |
| Post-intervention Trend Change (β_3_) | <0.01 | -0.02, 0.02 | 0.04 | -0.02, 0.10 | 0.01 | -0.02, 0.05 |

**B**

|  | **β** | **95% CI** | **β** | **95% CI** | **β** | **95% CI** |
| --- | --- | --- | --- | --- | --- | --- |
|  | **Full management zones**  **(*n* = 9)** | | **Town centre exempt zones**  **(*n* = 10)** | | **Time management zones**  **(*n* = 7)** | |
| Pre-intervention Intercept (β_0_) | <0.01 | 0.00, 0.30 | 0.13 | 0.01, 1.09 | 0.35 | 0.04, 2.37 |
| Pre-intervention Trend (β_1_) | 1.26 | 0.87, 2.85 | 1.05 | 0.88, 1.27 | 0.98 | 0.83, 1.16 |
| Post-intervention Level Change (β_2_) | 1.60 | 0.05, 123.06 | 0.03 | 0.00, 2.63 | 0.62 | 0.02, 12.94 |
| Post-intervention Trend Change (β_3_) | 0.76 | 0.33, 1.15 | 1.06 | 0.75, 1.78 | 1.07 | 0.86, 1.34 |
